# The effects of IL-17/IL-17R inhibitors on atherosclerosis in psoriasis and psoriatic arthritis: A protocol for systematic review and meta analysis

**DOI:** 10.1097/MD.0000000000024549

**Published:** 2021-02-12

**Authors:** Ningyuan Liu, Danni Su, Keshuai Liu, Binbin Liu, Shibo Wang, Xiaoyan Zhang

**Affiliations:** a Beijing University of Chinese Medicine; b Department of Dermatology and Venereology, China-Japan Friendship Hospital; c Peking University Health Science Center, Beijing; d Hangzhou Third Hospital, Hangzhou, China.

**Keywords:** biological agent, IL-17, psoriasis, psoriatic arthritis

## Abstract

**Background:**

Psoriasis (PSO) is a systemic inflammatory disorder that presents with erythematous scaling of the skin and is associated with autoimmune dysfunction. Atherosclerosis is one of the major comorbidities of PSO. PSO-associated inflammatory factor IL-17 could lead to vascular endothelial cell injury and atherosclerosis. While some research results show that IL-17 helps stabilize plaque formation. Efficacy and safety on PSO and psoriatic arthritis (PSA) of existing IL-17/IL-17R biologics (secukinumab, ixekizumab, brodalumab, and bimekizumab) have been clinically validated, but whether they can improve atherosclerotic outcomes in psoriatic patients remains controversial.

**Methods:**

Seven electronic search engines will be searched from inception to December 1, 2020, including PubMed, Embase, Scopus, PsycINFO, Global Health, Web of Science and the Cochrane Library. Clinical trial registries, potential grey literature, relevant conference abstracts, and reference lists of identified studies will also be searched. Literature selection, data extraction, and quality assessment will be done by 2 independent authors. Based on the heterogeneity test, the fixed effect or random effect model will be used for data synthesis. Changes in lung function will be evaluated as the primary outcome. Assessment of symptoms, quality of life, medication use, exacerbations and adverse events will be assessed as secondary outcomes. RevMan V. 5.3.5 (The Nordic Cochrane Centre, Copenhagen, Denmark) will be used for meta-analysis.

**Results:**

This study will provide a synthesis of current evidence of IL-17/IL-17R inhibitors on atherosclerosis in PSO and PSA.

**Conclusion:**

The conclusion of our study will provide updated evidence to judge whether IL-17/IL-17R inhibitors is an effective solution to atherosclerosis as comorbidity of PSO and PSA.

**PROSPERP registration number:**

CRD42020209897

## 1 Introduction

Psoriasis (PSO) is not only a chronic inflammatory skin disease, but also a chronic inflammatory systemic disease affecting nearly 0.91% to 8.5% of the world's population.^[1]^ More and more literature suggests that PSO, particularly severe disease, is associated with increased mortality^[2]^ and coexisting disease burden.^[3]^ PSO is an independent risk factor for atherosclerosis, patients with PSO have a higher incidence of subclinical atherosclerosis as indicated by coronary artery calcification score.^[4]^ Atherosclerosis is the main cause of Cardiovascular disease,which may be the most important cause of shortened life expectancy and increased mortality of PSO patients.^[5]^ Psoriatic arthritis (PSA) as a common comorbidity of PSO is also often associated with cardiovascular system diseases.

Atherosclerosis as a leading cause of cardiovascular disease, is considered to be the leading cause of death worldwide. Atherosclerosis is a chronic inflammatory state of the arterial wall in which the development and destabilization of plaques occur.^[6]^ Inflammation is the core mechanism of atherogenesis and endothelial dysfunction. Most of the studies support that IL-23/IL-17A axis may play an important role in various stages atherosclerotic, though some others not. IL-17 was found to be both inflammatory and protective in various inflammatory disease models, depending on the model and the environment in which it acts. At present, several clinical trials of IL-17/IL-17R inhibitor treating have been carried out. Some studies suggest an atherogenic role, while others suggest a protective role for atherosclerosis.^[7]^ Therefore, the function of IL-17 or IL-17R inhibitors on psoriatic patients with atherosclerosis remains controversial. This review is aim to systematically evaluate the effect of IL-17/IL-17R inhibitors on atherosclerosis in PSO and PSA.

## 2 Methods

### 2.1 Study registration

The protocol of this review has been registered on the international Prospective Register of Systematic Reviews (PROSPERO registration number: CRD42020209897) Available from: https://www.crd.york.ac.uk/prospero/

### 2.2 Inclusion criteria for study selection

#### 2.2.1 Types of studies

Inclusion criteria: Randomized controlled trials and observational studies that use IL-17/IL-17R inhibitors (secukinumab, ixekizumab, brodalumab, or bimekizumab) to treat PSO and/or PSA will be included in this study.

**Figure 1:**
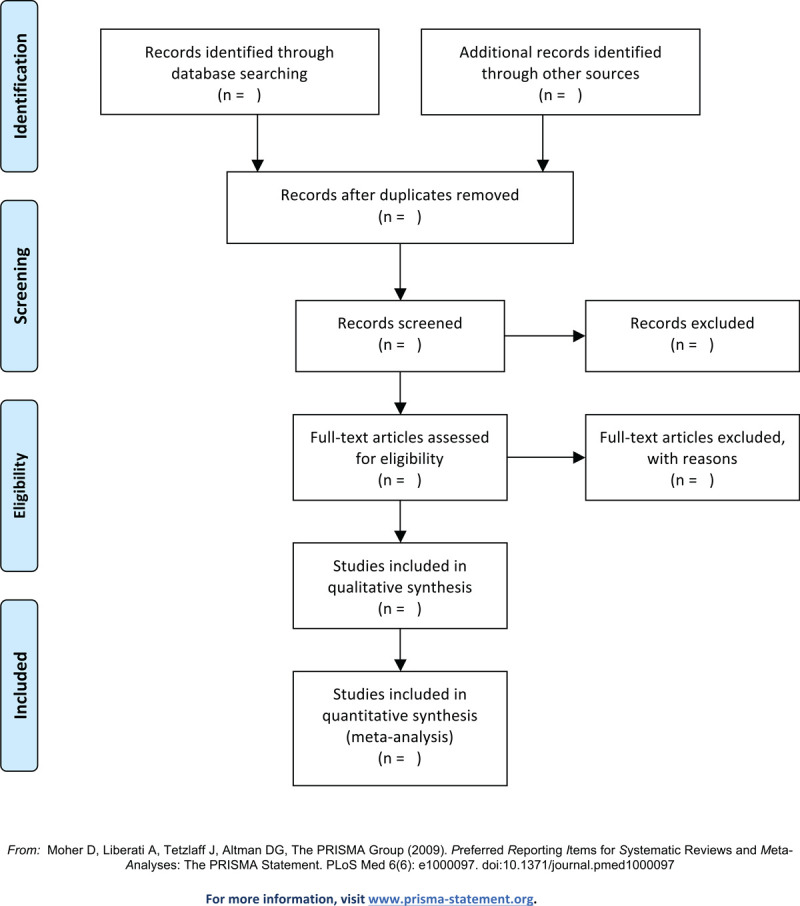
The preferred reporting items for systematic reviews and meta-analyses (PRISMA) flow chart of the selection process.

Exclusion criteria: participants do not meet the inclusion criteria; studies which have been published repeatedly; control group treated by other biological agents or system medicine (methotrexate, tyclosporine, tretinoine). The work flow of study selection is shown in Figure 1 following the PRISMA statement.^[8]^

#### 2.2.2 Types of participants

Inclusion criteria: Patients were required to have a formal diagnosis of PSO or PSA; PSO was clinically diagnosed and have PSO area severity index and/or body surface area scores in severity; PSA severity was measured by ACR20 and/or ACR50.

Exclusion criteria: a previous adverse event or lack of response to an IL-12/23 inhibitor that led to treatment discontinuation; diagnoses of other active skin conditions that may interfere with evaluation of PSO; use of any of the following PSO treatments: ultraviolet B phototherapy or topical prescription PSO treatments within 14 days of baseline, psoralen-ultraviolet A phototherapy within 30 days of baseline, oral PSO treatments within 30 days of baseline, biologics within 90 days of baseline (or 180 days for ustekinumab); use of investigational agents within 30 days or 5 half-lives (whichever is longer) of baseline; required oral or injectable corticosteroids; poorly controlled medical condition.

#### 2.2.3 Types of interventions

##### 2.2.3.1 Experimental interventions

We will include the antiIL-17/IL-17R biological agents (secukinumab, ixekizumab, brodalumab, and bimekizumab) with no limitations on the dose, the method of dosing or the duration of administration. In addition, studies with non-standard usage will be excluded.

##### 2.2.3.2 Control interventions

Comparisons interventions include placebo control or topical treatments. Besides, the following comparisons of studies will be excluded:

(1) comparing in other biological agents;
(2) comparing in system medicine;

#### 2.2.4 Types of outcome measures

##### 2.2.4.1 Primary outcomes

Atherosclerosis in PSO and PSA, which was measured by pulse wave velocity (PWV), flow-mediated dilation and artery plaque burden of carotid, femoral or coronary arteries.

##### 2.2.4.2 Secondary outcomes

Ankle brachial index, intima-media thickness, blood pressure, serum lipid: high density lipoprotein, low density lipoprotein, total cholesterol, triglycerides, high sensitivity c-reactive protein, homeostasis model assessment of insulin resistance.

### 2.3 Search methods for identification of studies

#### 2.3.1 Electronic sources

Seven electronic search engines will be searched from inception to December 1, 2020, including PubMed, Embase, Scopus, PsycINFO, Global Health, Web of Science, and the Cochrane Library. Medline's retrieval strategy (via PubMed) will be retrieved as shown in Table 1, and other electronic databases will be retrieved in the same manner.

**Table 1:** Search strategy for Medline (via PubMed).

Number	Search Items
1	Psoriasis
2	Psoriasis vulgaris
3	Psoriatic arthritis
4	Or/1–3
5	Secukinumab
6	Ixekizumab
7	Brodalumab
8	Bimekizumab
9	IL-17 inhibitor
10	IL-17a Inhibitor
11	IL-17R Inhibitor
12	Or/5–11
13	Cardiovascular disease
14	Artherosclerosis
15	Subclinical atherosclerosis
16	Endothelial dysfunction
17	Or/13–16
18	Randomized controlled trial
19	Controlled clinical trial
20	Trial
21	Randomized
22	Randomly
23	Or/18–22
24	4,12,17, and 23

#### 2.3.2 Searching other resources

We will check the references of the eligible studies and search the grey literature in OpenGrey. Conference abstracts will also be checked. The world health organization's international Clinical Trials registration platform and the Clinical Trials website clinicaltrials.gov will be searched for new reviews.

#### 2.3.3 Data collection and analysis

##### 2.3.3.1 Selection of studies

We will determine the basic process of inclusion according to the Cochrane Collaboration Reviewers’ Manual (5.1.0). An Excel spreadsheet will be used to record the study name, author, year, database, and whether the study met the eligibility criteria and should be included in the review. Reasons for inclusion and exclusion were also recorded in a spreadsheet during summary screening and full-text evaluation. The records in this spreadsheet will be used to generate the preferred report entries for the system review and meta-analysis flowchart (Fig. 1). The 2 reviews will independently execute all procedures and complete cross-checking. If any disagreement arises, the third author will be invited to assist in the discussion and decision. The GRADE profiler software (Version 3.6, The GRADE Working Group) will be used to determine evidence quality.

##### 2.3.3.2 Data extraction and management

We will make and pretest a standard collection form before data extraction. Two reviewers will independently extract data from selected studies and complete a data collection form. Differences and uncertainties will be resolved by consensus between the 2 review authors or by a third author to make a final decision. Included studies will be automatically numbered via EndNote. The following data will be focused:

1. Basic information: title, citation, first and corresponding author, journal, type, year of publication, and contact information of the included study;
2. Methods: study design, sample size, randomization, allocation concealment, blinding, inclusion criteria, and exclusion criteria;
3. Participants: age, gender, occupation, race, region, and comorbidities;
4. Interventions: name of biological agent, route of administration, dose, start date, and duration;
5. Results: primary outcomes including PWV (PWV = distance/Δtime [m/s]), flow-mediated dilation [(peak diameter minus baseline diameter)/baseline diameter] × 100 and artery plaque burden; secondary outcomes including ankle brachial index (index), blood pressure (mm/Hg), high density lipoprotein (mg/dL), low density lipoprotein (mg/dL), total cholesterol (mg/dL),TC (mg/dL), high sensitivity c-reactive protein (mg/dL), homeostasis model assessment of insulin resistance (index); adverse events;
6. Additional Information: funding agency, conflict of interest, ethics committee approval, sample size calculation included;

##### 2.3.3.3 Assessment of I^2^

Two investigators will assess the risk of bias using the “Risk of Bias” tool of the Cochrane Handbook (V.5.3.5). The authors will judge whether the random allocation sequence is correctly generated; whether the allocation scheme concealment is perfect; whether the blind method is perfect; whether the result data are complete; whether the study report indicates that there is no selective reporting of results and whether the study has other factors that cause a high risk of bias; When there is any disagreement, it will be resolved through discussion or consultation with the third author.

##### 2.3.3.4 Measures of treatment effect

The continuous data will be expressed as mean difference or standard mean difference with 95% confidence intervals (CIs), and the dichotomous variable will be estimated by the risk ratio with 95% CIs. Ordinal data was first grouped in rank order and converted to dichotomous variable or continuous variable for analysis after completion of all study data extraction. Adverse reactions will be assessed and tabulated by descriptive techniques.

##### 2.3.3.5 Dealing with missing data

The reviewer will contact the first or corresponding author of the study by telephone, email, or mail to collect missing data.

##### 2.3.3.6 Assessment of heterogeneity

The heterogeneity will be analyzed by Chi-squared test (a = 0.1), I^2^ < 50% will be considered to contain small heterogeneity, while I^2^ > 50% will be considered to have greater heterogeneity. Sensitivity analysis will be used to detect stability and reliability of the results. In case of sufficient sample size, subgroup analysis will be carried out among different biological agent types or age groups.

##### 2.3.3.7 Assessment of reporting bias

If there 10 or more studies are included, then a funnel plot will be used to detect report bias.

##### 2.3.3.8 Data synthesis

Data analysis and synthesis will be performed using RevMan V.5.3.5 (The Nordic Cochrane Centre, Copenhagen, Denmark). Continuous data are expressed as MD/standard mean difference, 95% CIs, and the outcome of the 2 classifications are expressed as RR, 95% CIs. When I^2^ < 50%, the fixed effect model is used for analysis; when I^2^ > 50%, the random effect model is selected. When significant clinical heterogeneity is observed, researchers can turn to subgroup or sensitivity analysis, or just descriptive analysis. a = 0.05 was used to evaluate the meta-analysis. If quantitative synthesis is not appropriate we will not perform a meta-analysis and data will be presented descriptively.

##### 2.3.3.9 Subgroup analysis

Subgroup analyses will be performed according to different interventions, control measures, and outcome indicators. Adverse reactions will be assessed and tabulated by descriptive techniques. (The number of studies is > 10.)

## 3 Discussion

IL-17 is a proinflammatory cytokine that may play a dual role in the cardiovascular system in promoting inflammation and promoting arterial plaque stabilization in a complex cytokine network. Four IL-17/IL-17R inhibitors have been approved for the treatment of PS and PSA, with good efficacy and safety in clinical practice.

Animal and clinical experimental studies have shown that IL-17 has a protective effect on atherosclerosis, but there is also recent evidence to support that IL-17 has an atherogenic effect. Therefore, the function of IL-17 remains controversial and awaits more direct studies to address the question. In the short term, IL-17/IL-17R inhibitors do not significantly increase the risk of major adverse cardiovascular events, but whether they improve vascular endothelial cell function and prevent the progression of cardiovascular comorbidity remains controversial, existing clinical trial results are not sufficient to support clinical decisions, and more relevant clinical trials are ongoing. There are no systematic reviews and meta-analyses addressing the effects of IL-17/IL-17R inhibitors on atherosclerosis and vascular endothelial cell function. We hope that this will be summarized and concluded by the systematic review. However, there may be some potential pitfalls in systematic reviews.

## Author contributions

**Conceptualization:** Ningyuan Liu, Xiaoyan Zhang.

**Data curation:** Danni Su, Keshuai Liu, Binbin Liu.

**Formal analysis:** Ningyuan Liu,Danni Su, Keshuai Liu.

**Methodology:** Ningyuan Liu, Shibo Wang.

**Project administration:** Ningyuan Liu, Xiaoyan Zhang.

**Supervision:** Xiaoyan Zhang.

**Writing – original draft:** Ningyuan Liu.

**Writing – review & edting:** Ningyuan Liu, Xiaoyan Zhang.

## Abbreviations

Abbreviations: CIs = confidence intervals, PSA = psoriatic arthritis, PSO = psoriasis, PWV = pulse wave velocity.
